# ERK-Mediated Activation of Fas Apoptotic Inhibitory Molecule 2 (Faim2) Prevents Apoptosis of 661W Cells in a Model of Detachment-Induced Photoreceptor Cell Death

**DOI:** 10.1371/journal.pone.0046664

**Published:** 2012-09-28

**Authors:** Cagri G. Besirli, Qiong-Duon Zheng, David M. Reed, David N. Zacks

**Affiliations:** Department of Ophthalmology and Visual Sciences, W.K. Kellogg Eye Center, University of Michigan, Ann Arbor, Michigan, United States of America; Thomas Jefferson University, United States of America

## Abstract

In this study, we examined the role of Fas apoptotic inhibitory molecule 2 (Faim2), an inhibitor of the Fas signaling pathway, and its regulation by stress kinase signaling during Fas-mediated apoptosis of 661W cells, an immortalized photoreceptor-like cell line Treatment of 661W cells with a Fas-activating antibody led to increased levels of Faim2. Both ERK and JNK stress kinase pathways were activated in Fas-treated 661W cells, but only the inhibition of the ERK pathway reduced the levels of Faim2. Blocking the ERK pathway using a pharmacological inhibitor increased the susceptibility of 661W cells to Fas-induced caspase activation and apoptosis. When the levels of Faim2 were reduced in 661W cells by siRNA knockdown, Fas activating antibody treatment resulted in earlier and more robust caspase activation, and increased cell death. These results demonstrate that Faim2 acts as a neuroprotectant during Fas-mediated apoptosis of 661W cells. The expression of Faim2 is triggered, at least in part, by Fas-receptor activation and subsequent ERK signaling. Our findings identify a novel protective pathway that auto-regulates Fas-induced photoreceptor apoptosis in vitro. Modulation of this pathway to increase Faim2 expression may be a potential therapeutic option to prevent photoreceptor death.

## Introduction

Separation of outer retina from the retinal pigment epithelium (RPE) is a common form of injury that may occur alone in retinal detachment or with other pathologic processes in blinding diseases such as age-related macular degeneration or diabetic retinopathy. Despite significant advances in the medical and surgical management of retina-RPE separation, patients often lose vision, primarily due to the death of photoreceptors [1], [2]. Our previous studies demonstrated that the main pathologic event causing photoreceptor death is the activation of the apoptotic Fas signaling and the downstream cascade of caspases 8, 3, 7 and 9 [3], [4], [5]. Preventing Fas pathway activity provides significant protection against separation-induced death of the photoreceptors.

Experimental data from animal models show that despite rapid activation of apoptosis after retina-RPE separation, a significant number of photoreceptors survive for extended periods of time [4], [5]. The clinical correlation of this experimental observation is that patients with retinal detachments affecting central vision generally recover near-normal vision if the detachment is repaired within one week [6], [7]. If repair is delayed beyond one week, visual outcomes become significantly poorer. These experimental and clinical observations suggest that early in the course of retinal detachment, anti-apoptotic pathways are activated within the retina to counteract the effect of pro-apoptotic signals and that they are responsible for the visual outcome related window-of-opportunity for reattachment. We have shown the significance of two such pathways in the retina, activation of IL-6 signaling and detachment-induced increase in autophagy [8], [9].

Our gene microarray analysis of experimental detachments in rats revealed increased expression of genes involved in Fas-receptor signaling and stress-response pathways [10]. One gene of significance that emerged from that study was one coding for Fas apoptotic inhibitory molecule 2 (Faim2). Faim2 is an evolutionarily conserved protein and is predominantly expressed in neuronal cells as a 35 kDa membrane protein. Faim2 belongs to a larger group of evolutionary conserved anti-apoptotic proteins known as the Lifeguard (LFG) family [11]. Faim2 was shown to prevent apoptosis by direct interaction with Fas upstream of Fas-associated death domain containing protein (FADD) [12]. Faim2 expression in cerebellar granule neurons increases their resistance to Fas mediated apoptosis [13]. Neurons of Faim2-deficient mice are more susceptible to combined oxygen-glucose deprivation in vitro and caspase-associated cell death and neurological impairment after cerebral ischemia *in vivo* [14]. Similarly, Faim2 is required for the development and survival of granular and Purkinje cells [15].

Another set of genes that were upregulated in our microarray analysis of experimental detachments in rats were downstream targets of Mitogen-Activated Protein Kinase (MAPK) superfamily [10]. The MAPK super-family is composed of three major sets of kinases: the extracellular-receptor kinases (ERK), the c-Jun N-terminal kinases (JNK) and the p38 MAPKs [16]. Evidence from neuronal injury models indicates that stress kinase signaling is involved in Fas-receptor activation [17], [18]. Members of the MAPK super-family have been shown to be critical for cell survival as well as cell death in models of apoptotic and non-apoptotic cell death and their role largely depends on the context and cellular insult.

In this study, we tested the hypothesis that increased Faim2 expression prevents photoreceptor apoptosis. We first analyzed Faim2 expression and MAPK signaling during photoreceptor apoptosis using our well-established in vivo model of experimental retinal detachment. To further characterize the role of Faim2 as a survival protein, we examined Fas-induced apoptosis in 661W cells, an in vitro model of retinal photoreceptors. Our results demonstrate that retinal detachment increases Faim2 protein levels in vivo, and this finding is reproduced in vitro by exogenous activation of Fas signaling in 661W cells. Fas-signaling also leads to increased activity of ERK and JNK kinases in 661W cells. The expression of Faim2 is regulated by ERK signaling as pharmacological inhibition of ERK activity reduces Faim2 levels. The inhibition of ERK signaling results in more robust activation of caspases and leads to increased Fas-mediated 661W cell death. We propose that this effect is mediated, at least in part, by decreasing levels of Faim2 in cells. These data provide compelling evidence for the critical role of Faim2 in the auto-regulation of Fas-mediated apoptosis in photoreceptor cells.

## Materials and Methods

### Ethics Statement

All experiments were performed in accordance with the Association for Research in Vision and Ophthalmology (ARVO) Statement for the Use of Animals in Ophthalmic and Vision Research. The protocol was approved by the University Committee on Use and Care of Animals of the University of Michigan (Protocol number: 08553). All surgery was performed under ketamine and xylazine anesthesia, and all efforts were made to minimize suffering.

### Experimental Model of Retinal Detachment

Detachments were created in adult male Brown-Norway rats (300–400 g) (Charles River Laboratories, Wilmington, MA) as previously described [3]. Pupils were dilated with topical phenylephrine (2.5%) and tropicamide (1%). A 20-gauge microvitreoretinal blade was used to create a sclerotomy located 2 mm posterior to the limbus with care taken to avoid lens damage. A Glaser subretinal injector (32-gauge tip; BD Ophthalmic Systems, Franklin Lakes, NJ) was introduced through the sclerotomy into the vitreous cavity and then through a peripheral retinotomy into the subretinal space. Sodium hyaluronate (10 mg/mL) (Abbott Medical Optics, Healon OVD) was slowly injected to detach the neurosensory retina from the underlying retinal pigment epithelium. In all experiments, approximately one-third to one-half of the neurosensory retina was detached. Detachments were created in the left eye. The right eye served as the control, with all the steps of the procedure performed, except for introduction of the subretinal injector and injection of the sodium hyaluronate.

### Cell Culture

The 661W photoreceptor cell line was generously provided by Dr. Muayyad Al-Ubaidi (Department of Cell Biology, University of Oklahoma Health Sciences Center, Oklahoma City, OK, USA) [19]. The 661W cell line was maintained in Dulbecco's modified Eagle's medium containing 10% fetal bovine serum, 300 mg/L glutamine, 32 mg/L putrescine, 40 µL/L of β-mercaptoethanol, and 40 µg/L of both hydrocortisone 21-hemisuccinate and progesterone. The media also contained penicillin (90 units/mL) and streptomycin (0.09 mg/mL). Cells were grown at 37°C in a humidified atmosphere of 5% CO_2_ and 95% air.

### Western Blot Analysis

Retinas from experimental eyes with detachments and control eyes without detachments were dissected from the RPE-choroid, homogenized, and lysed in buffer containing 10 mM HEPES (pH 7.6), 0.5% IgEPal, 42 mM KCl, 1 mM phenylmethylsulfonyl fluoride (PMSF), 1 mM EDTA, 1 mM EGTA, 1 mM dithiothreitol (DTT), 5 mM MgCl_2_, and 1 tablet of protease inhibitors per 10 mL buffer (Complete Mini; Roche Diagnostics GmbH, Madison, WI). 661W cells were lysed and homogenized in lysis buffer (as above). The homogenates were incubated on ice and centrifuged at 22,000×g at 4°C for 60 min. The protein concentration of the supernatant was then determined (DC Protein Assay kit; Bio-Rad Laboratories, Hercules, CA). The protein samples were separated on SDS-polyacrylamide gels (Tris-HCl Ready Gels; Bio-Rad Laboratories, Hercules, CA). After electrophoretic separation, the proteins were transferred onto polyvinylidene fluoride (PVDF) membranes (Immobilon-P; Millipore, Billerica, MA). Protein bands were visualized with Ponceau S staining, and the lanes were assessed for equal loading by densitometry of entire lanes. Membranes were then immunoblotted with antibodies according to the manufacturer's instructions. The following antibodies were used: Faim2/LFG (Anaspec, Fremont, CA), P-JNK, JNK, P-ERK, ERK, P-c-Jun (Cell Signaling, Danvers, MA), Actin (Santa Cruz Biotechnology, Santa Cruz, CA). Densitometry measurements were performed using Image J software [20].

### Caspase Assays

Caspase 8 and caspase 3/7 activities were measured using luminescent tetrapeptide cleavage assay kits (Promega, Madison, WI). The 661W cells were seeded in 96-well plates (Nunc, Rochester, NY) at 1000–1500 cells/well for 24 h prior to treatment or siRNA transfection. Cells were treated with 500 ng/mL of Fas-agonistic Jo2 monoclonal antibody (BD Biosciences, Franklin Lakes, NJ). In some experiments, cells were pretreated with MEK inhibitor U0126, JNK inhibitor SP600125, or DMSO. Caspase activity was measured at 12 and 24 h after treatment by incubating the cells with the pro-luminescent substrate in 96-well plates following manufacturer's instructions. Controls included untreated cells and wells with no cells. Luminescence was measured in a plate reader luminometer (Turner Biosystems, Sunnyvale, CA).

### Cell Viability

Cell viability was measured with a luminescent assay kit (Promega, Madison, WI). The 661W cells were seeded in 96-well plates (Nunc, Rochester, NY) at 1000–1500 cells/well for 24 h prior to treatment. Cells were treated with 500 ng/mL of Fas-agonistic Jo2 monoclonal antibody (BD Biosciences, Franklin Lakes, NJ). In some experiments, cells were pretreated with MEK inhibitor U0126, JNK inhibitor SP600125, or DMSO. Cell viability was measured at 48 and 72 h after treatment by incubating the cells with the pro-luminescent substrate in 96-well plates following manufacturer's instructions. Controls included untreated cells and wells with no cells. Luminescence was measured in a plate reader luminometer (Turner Biosystems, Sunnyvale CA).

### siRNA treatment

661W cells were treated with small inhibitory RNAs against mouse Faim2 (siFaim2, Invitrogen Life Technologies, Carlsbad, California) to prevent Fas-induced increase in the transcription of Faim2 gene. siFaim2 or control siRNA (labeled with Cy3) were transfected into cells using Lipofectamine RNAiMax (Invitrogen Life Technologies, Carlsbad, California) according to manufacturer's instructions. siRNA transfection efficiency was determined by direct examination of Cy3-labeled control siRNA uptake in cells using an inverted fluorescent microscope. Cells were treated 36–48 h after siRNA transfection.

### Data Analysis

Statistical analysis comparing treatment groups was performed using two-tailed Student's t-tests assuming equal variance (SigmaPlot 11, Systat Software, San Jose, CA). Differences were considered significant for p<0.05.

## Results

### Retinal Detachment induces Faim2 expression

**Figure 1:**
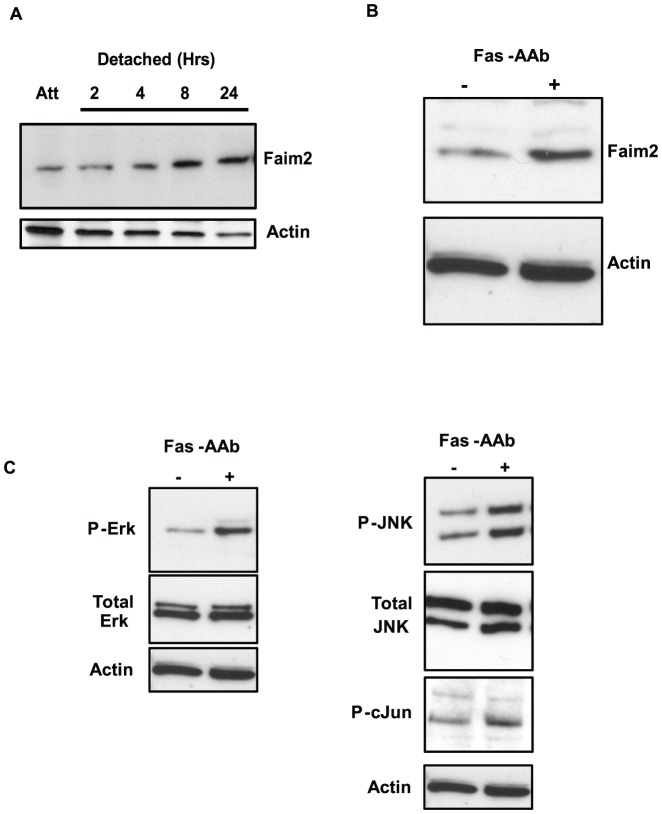
Photoreceptor stress leads to Fas-dependent increase in Faim2 and MAPK expression. A) Western blots showing increased levels of Faim2 as a function of time after retina-RPE separation. Actin is shown as loading control. As expected, the actin level decreases as a function of time after detachment due to the retraction of the photoreceptor outer segment after separation of the retina from the retinal pigment epithelium[26]. Att: Attached. B) 661W cells treated with 500 ng/mL of Fas-activating antibody (Fas-AAb, +) show increased levels of Faim2 protein. Cells were treated for 4 hours, lysed and protein expression was analyzed via immunoblotting. Control cells were not treated with Fas-AAb (-). Actin is shown as loading control. C) Activation of ERK and JNK signaling was analyzed in 661W cells treated with 500 ng/mL of Fas-activating antibody (Fas-AAb) by immunoblotting. ERK signaling was evaluated by phospho-p44/42 (P-ERK) antibody and JNK signaling by measuring levels of phospho-JNK (P-JNK) and phospho-c-Jun (P-c-Jun) after 4 hours of Fas-AAb treatment. Levels of total ERK and total JNK remained stable, suggesting specific conversion of existing kinases to their phosphorylated form. Actin is shown as loading control. Similar results were obtained in two independent trials.

In our microarray gene expression study of retinal detachment, we found that Faim2 was induced at 24 h after retina-RPE separation. To confirm the microarray data, we evaluated the levels of Faim2 protein in detached rat retinas. We observed that levels of Faim2 started to increase within 4 h of retinal detachment (Figure 1A) and peaked by 24 h. This increase in Faim2 expression persisted to the latest time point tested (7 days, data not shown). This finding confirmed our microarray data that retinal detachment leads to increased levels of Faim2.

### Fas signaling leads to elevated Faim2 expression in 661W cells

We next determined whether the Faim2 increase seen in our in vivo system of experimental retinal detachment could be modeled using our in vitro assay of Fas-mediated photoreceptor apoptosis; the primary pathway leading to photoreceptor death during retinal detachment. The 661W cell line is a photoreceptor line that has been immortalized by the expression of SV40-T antigen under control of the human interphotoreceptor retinol-binding protein (IRBP) promoter [19]. 661W cells express cone photoreceptor markers, including blue and green cone pigments, transducin, and cone arrestin [21]. We previously showed that treatment of 661W cells with an antibody that activates the Fas-receptor (Fas-activating antibody or Fas-AAb) leads to caspase 8 activation and apoptosis, confirming that Fas death receptor signaling is intact in 661W cells [8]. When we treated 661W cells with Fas-AAb, Faim2 expression increased significantly (Figure 1B), demonstrating that similar to our in vivo results in the experimental retinal detachment, direct activation of Fas receptor signaling in 661W photoreceptors leads to elevated Faim2 levels.

### 661W cells show increased ERK and JNK signaling following Fas pathway activation

Our previous studies showed increased ERK and JNK signaling in detached rodent retinas as detected by increased levels of phosphorylated forms of these stress kinases [10]. We now sought to determine whether exogenous activation of Fas signaling could lead to MAPK activation in 661W cells. When 661W cells were treated with Fas-AAb, both ERK and JNK phosphorylation increased significantly (Figure 1C). The levels of total ERK and JNK remained stable after Fas-AAb treatment, indicating that Fas signaling induces conversion of these stress kinases from the existing inactive form to phosphorylated active form. Further evidence of JNK activation was demonstrated by increased phosphorylation of one of its downstream targets, transcription factor c-Jun (Figure 1C).

### Inhibition of stress kinases enhances Fas-mediated apoptosis of 661W cells

**Figure 2:**
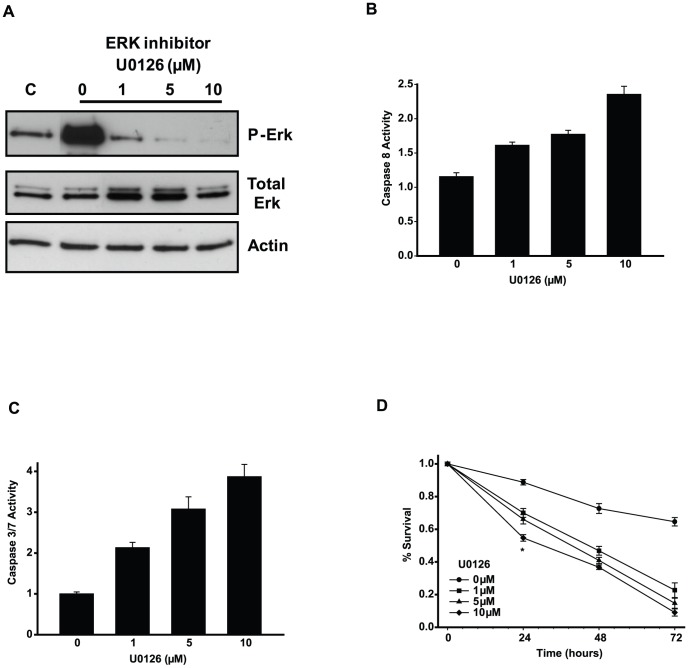
Inhibition of ERK signaling increases Fas-mediated caspase activation and 661W cell apoptosis. A) Cells were pretreated with U0126 1 h prior to Fas-AAb addition. Protein was isolated 4 h later and levels of P-ERK and total ERK were determined by immunoblotting. C, untreated controls (No Fas-AAb). Actin is shown as loading control. Similar results were obtained in three independent trials. B) Activation of caspase 8 was evaluated in the presence of U0126 after 24 h of Fas-AAb treatment, n = 16–24, mean±SE. C) Activation of caspase 3/7 was evaluated in the presence of U0126 after 24 hours of Fas-AAb treatment, n = 16–24, mean±SE. D) The effect of ERK inhibitor on Fas-mediated photoreceptor apoptosis was determined in 661W cells, n = 16–24, mean±SE, *p<0.05. Data are representative of 2–3 independent trials.

**Figure 3:**
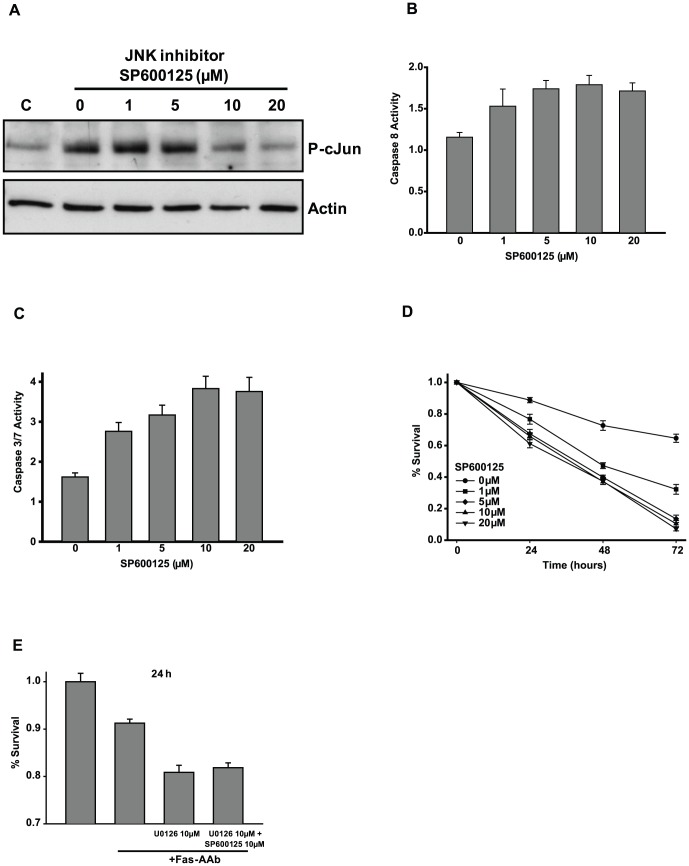
JNK signaling is anti-apoptotic during Fas-mediated 661W cell death. A) Cells were pretreated with SP600125 1 hour prior to Fas-AAb addition. Protein was isolated 4 h later and levels of P-c-Jun were determined by immunoblotting. C, untreated controls (No Fas-AAb). Actin is shown as loading control. Similar results were obtained in three independent trials. B) Activation of caspase 8 was evaluated in the presence of SP600125 after 24 h of Fas-AAb treatment, n = 16–24, mean±SE. C) Activation of caspase 3/7 was evaluated in the presence of SP600125 after 24 h of Fas-AAb treatment, n = 16–24, mean±SE. D) The effect of JNK inhibitor on Fas-mediated photoreceptor apoptosis was determined in 661W cells, n = 16–24, mean±SE. Data are representative of 2–3 independent trials. E) The effect of simultaneous inhibition of JNK and ERK pathways on 661W cell death, n = 8, mean±SE.

MAPKs play different roles during cellular apoptosis depending on the cell's molecular context. In general, ERK signaling is important for activating pro-survival signals, whereas JNK signaling tends to be pro-apoptotic. To test the role of ERK and JNK signaling, we used pharmacological inhibitors of ERK and JNK phosphorylation. To reduce ERK activity, we inhibited its upstream activator, ERK kinase MEK, with U0126. JNK signaling was reduced by a direct JNK inhibitor, SP600125. We found that U0126 was effective in preventing the Fas-AAb-induced phosphorylation of ERK starting at low doses, with maximal inhibition at 10 µM (Figure 2A). Similarly, SP600125 blocked Fas-AAb-dependent phosphorylation of JNK-target c-Jun, with maximal effect seen at the 10–20 µM concentration level (Figure 3A).

We next determined the effect of blocking MAPK signaling on Fas-mediated apoptosis of 661W cells. When cells were exposed to Fas-AAb in the presence of U0126, there was a dose-dependent increase in caspase 8 and caspase 3/7 activity (Figure 2B,C). Similarly, U0126 treatment increased Fas-AAb-mediated cell death (Figure 2D). This dose dependence was consistent with the dose of inhibitor found to block ERK phosphorylation seen with immunoblotting. Similar findings were observed with inhibition of the JNK pathway. SP600125 increased caspase 8 and caspase 3/7 activities in a dose-dependent manner (Figure 3B,C). 661W cells were more sensitive to Fas-mediated cell death when JNK signaling was blocked with SP600125 (Figure 3D). These findings strongly indicate that both ERK and JNK signaling pathways are important in activating survival pathways in 661W cells undergoing Fas-mediated apoptosis. The sensitivity of 661W cells to apoptotic stimulus was similar when inhibitors against ERK pathway alone or ERK and JNK pathways together were used (Figure 3E), demonstrating that the effect of inhibiting these pathways on cell survival was not additive.

### Faim2 expression is regulated by ERK signaling

**Figure 4:**
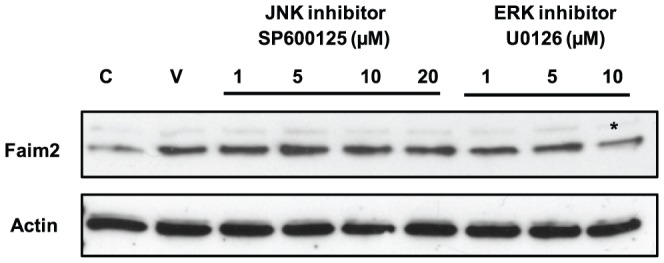
Faim2 expression is regulated by ERK signaling. 661W cells were treated with Fas-AAb with or without MAPK inhibitors and levels of Faim2 were determined by immunoblotting. The JNK inhibitor SP600125 did not affect Faim2 levels, while the ERK inhibitor U0126 showed a dose-dependent inhibition of the increase in Faim2 levels normally seen after treatment with the Fas-activating antibody (*). C, untreated controls (No Fas-AAb); V, vehicle only control (DMSO). Similar results were obtained in three independent trials.

Based on the results of MAPK inhibitor experiments, we hypothesized that ERK or JNK pathway may be regulating photoreceptor survival by controlling Faim2 levels after Fas-receptor activation. To answer this question, we analyzed Faim2 expression in 661W cells when they were treated with Fas-AAb in the presence of MAPK inhibitors. Inhibition of the ERK pathway with U0126 reduced the levels of Faim2 in 661W cells (Figure 4). In contrast, JNK inhibitor SP600125 did not show any effect on Faim2 expression in Fas-treated cells (Figure 4). These results demonstrate that ERK signaling is important in upregulation of Faim2 during 661W cell apoptosis, whereas JNK pathway has no detectable effect.

### Reduced Faim2 expression enhances Fas-mediated 661W cell death

**Figure 5:**
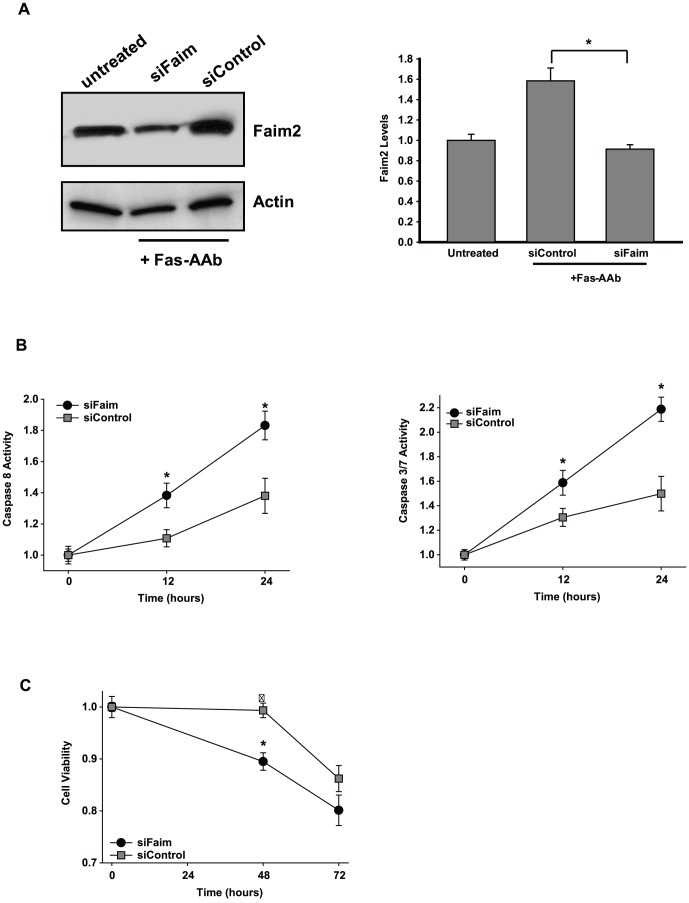
Faim2 expression inhibits Fas-mediated apoptosis. A) siFaim reduced Faim2 protein levels in 661W cells. Cells were transfected with siFaim or siControl and treated with 500 ng/ml of Fas-AAb after 36 h. Levels of Faim2 were determined by immunoblotting after 4 h of Fas-AAb treatment. B) Mean densitometry of data from 4 independent Western blots, Faim2 levels were normalized to the actin levels, n = 4, mean±SE, *p<0.05. C, D) Caspase 8 and 3/7 activities were increased in 661W cells after Faim2 knockdown. Cells were treated with Fas-AAb 36 h after siFaim transfection. Caspase activity was determined at 12 and 24 h. n = 14–16, mean±SE, *p<0.05. E) Fas-mediated photoreceptor cell death is accelerated after Faim2 knockdown. 661W cells were treated with Fas-AAb 36 h after siFaim transfection. Cell viability was determined at 48 and 72 h. n = 22–24, mean±SE, *p<0.05. Data is representative of 2–3 independent trials.

To test the hypothesis that the increase in Faim2 levels acted as an inhibitor of apoptosis, we performed a loss-of-function experiment. We used small inhibitory RNA (siRNA) to reduce Faim2 expression levels in 661W cells. 661W cells transfected with siFaim showed reduced levels of Faim2 protein after Fas-AAb treatment (Figure 5A,B). This decline in protein expression was not seen in cells transfected with the control siRNA (siControl). Next, the effect of Faim2 knockdown on Fas-mediated photoreceptor apoptosis was evaluated. In siFaim transfected cells, Fas-AAb treatment resulted in more robust caspase 8 activation compared with cells transfected with control siRNA (Figure 5C). This caspase activation occurred earlier and peaked at a significantly higher level when Faim2 expression was reduced in 661W photoreceptor cells with siFaim. Similarly, caspase 3/7 activity was more robust in cells with reduced levels of Faim2 (Figure 5D). These results demonstrate that Faim2 acts as an anti-apoptotic factor in 661W cells and Faim2 function is critical for blocking early caspase activation.

We next determined the effect of reducing Faim2 levels on Fas-mediated apoptosis of 661W cells. When cells were exposed to Fas-AAb after siFaim transfection, apoptosis occurred at an earlier time point (Figure 5E). These findings strongly indicate that Faim2 acts as a neuroprotectant during Fas-mediated apoptosis of 661W cells.

## Discussion

**Figure 6:**
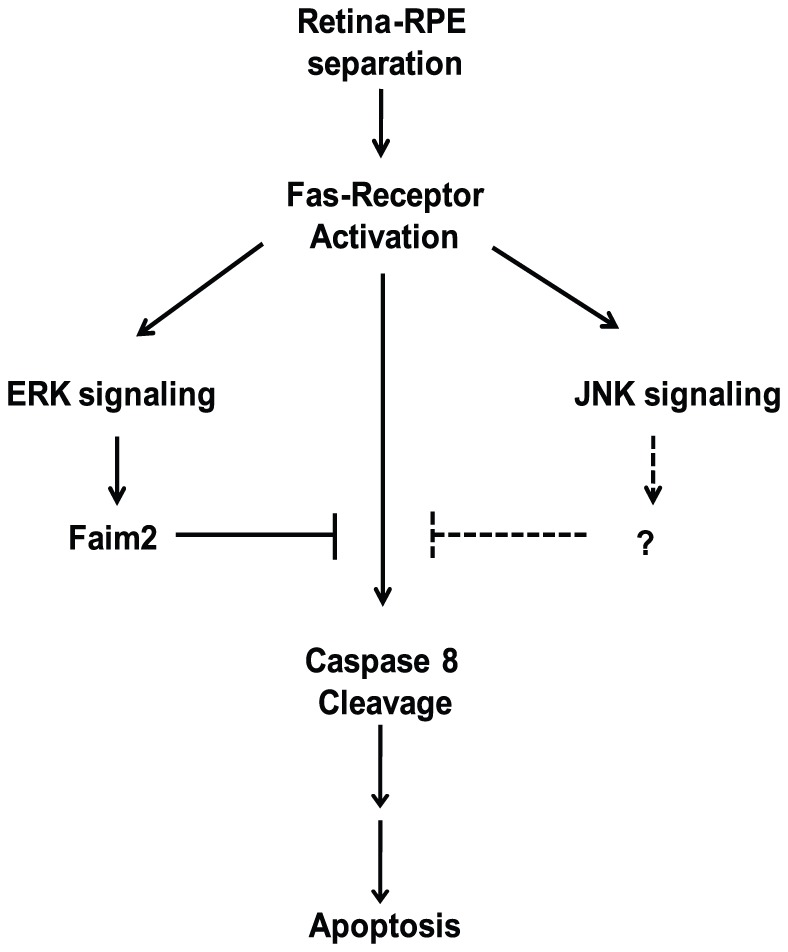
Novel model of the auto-regulation of Fas-mediated photoreceptor apoptosis via ERK activation and increased production of Faim2.

In this study, we show that Faim2 acts as a neuroprotectant via Fas-mediated ERK pathway signaling. Blocking ERK pathway reduces Faim2 levels and increases caspase activity and 661W cell death after Fas receptor activation. As previously mentioned, Fas signaling is critical for photoreceptor apoptosis after retinal detachment. A novel finding demonstrated by this study is that direct activation of Fas receptor causes rapid increase in Faim2 levels in photoreceptor cells in vivo and in vitro. This reveals a new autoregulatory mechanism of apoptotic Fas receptor signaling (Figure 6).

We have previously shown that the activation of the Fas death receptor signaling is the main event causing photoreceptor apoptosis after the separation of the neurosensory retina from the underlying RPE [3], [4], [5]. Molecular or pharmacological interventions that prevent Fas receptor activation or transcription of new Fas receptor provide significant protection against the separation-induced death of the photoreceptors [5], [8]. Although the biochemical markers of Fas activation are detected shortly after retina-RPE separation, significant numbers of photoreceptors can survive for extended periods of time. This observation led us to hypothesize that in addition to apoptotic pathways, survival pathways are activated in photoreceptors that are detached from the underlying RPE. Consistent with this hypothesis, gene transcription profile within the retina after photoreceptor-RPE separation reveals several candidate genes that may act as retinal neuroprotectants. We recently showed that IL-6 is necessary for the survival of photoreceptors after detachment from the RPE [9]. We also found that retinal detachment resulted in Fas dependent activation of autophagy in photoreceptors, and autophagy acted as a pro-survival pathway through inhibition of apoptosis [22]. Further studies are needed to determine whether Faim2, IL-6 and autophagy are part of the same survival pathway or act as independent, parallel survival signals.

The data presented in this report strongly argue that Faim2 functions as an anti-apoptotic molecule during Fas-mediated cell death. Faim2 is an evolutionarily conserved protein and predicted to have seven transmembrane domains and a small cytoplasmic domain at the N-terminus [23]. Faim2 appears to act at one of the early steps of Fas receptor death signaling to exert its survival effect. Co-immunoprecipitation assays showed that overexpressed Faim2 interacts with Fas receptor directly, but it does not bind downstream effectors of Fas signaling such as FADD or interfere with binding of Fas agonists [12]. However, the exact mechanism of how Faim2 inhibits Fas receptor signaling under physiologic conditions is unclear. Further experiments are needed to determine if Fas-Faim2 interaction occurs in neuronal cells undergoing Fas-mediated apoptosis. Our knockdown experiments with siRNA against Faim2 demonstrate that caspase 8 activation is regulated by Faim2. Since caspase 8 cleavage is a proximal event in Fas death receptor signaling, this knockdown data would be consistent with the model of direct Faim2-Fas receptor interaction. Another question remaining to be answered is how ERK increases Faim2 protein levels in cells. Whether ERK regulates Faim2 expression through increased gene transcription, stabilizing Faim2 mRNA, or slowing down Faim2 protein degradation needs to be characterized.

Inhibition of the ERK signaling pathway by U0126 increased the susceptibility of photoreceptor cells to Fas activation at a dose required to inhibit Faim2 expression, but lower concentrations of U0126 which did not have any detectable effect on Faim2 levels, the cells were still more vulnerable to apoptotic signaling. We believe that this data is consistent with Faim2 as one of the survival proteins activated by ERK and the role of Faim2 is only partially responsible for ERK-mediated photoreceptor protection. Our data indicates that there are other ERK-dependent survival proteins in the cell in addition to Faim2. Another possible explanation is the possible role of ERK signaling on Faim2 post-translational regulation, such as phosphorylation. It is possible that inhibition of ERK signaling with U0126 at lower doses of 1 or 5 µM is sufficient to block Faim2 phosphorylation and acitivity, but higher concentrations of U0126 is required to see any effect of protein levels on Western blots. Additional studies examining the possible role of ERK in Faim2 phosphorylation and its potential role on protein function or stability are needed.

In addition to ERK, the induction of Fas receptor signaling in photoreceptor cells activated JNK stress-kinase pathway. While ERK signaling appeared to be important in regulating Faim2 expression, inhibiting JNK activity with SP600125 did not have any effect on Faim2 levels. However, JNK inhibition in Fas-treated cells caused increased caspase activation and cell death, indicating that JNK activity is an important survival mechanism in photoreceptors after Fas exposure. Anti-apoptotic molecules regulated by JNK signaling in photoreceptors remain to be identified. One possibility is that JNK regulates Faim2 function by phosphorylating it. The analysis of mouse Faim2 amino acid sequence reveals a potential JNK phosphorylation target located in the C-terminus between predicted transmembrane domains 6 and 7 (amino acid 286) [24]. This sequence motif is evolutionary conserved among different mammalian and non-mammalian species [23]. We are currently investigating the possibility of JNK-mediated Faim2 phosphorylation as a mechanism of regulating Faim2 function and photoreceptor apoptosis.

Another possible mechanism of JNK-induced photoreceptor survival may involve the autophagy pathway. We previously showed that autophagy acts as a survival signal in photoreceptors after Fas activation [22]. JNK-1 phosphorylates Bcl-2, which allows Beclin-1 to dissociate from Bcl-2 and activate autophagy [25]. In the presence of JNK inhibition, Bcl2/Beclin1 complex would remain stable and activation of photoreceptor autophagy would be impaired, making the cells more susceptible to Fas-induced death.

In summary, we describe a novel autoregulatory mechanism of Fas receptor signaling during 661W cell apoptosis. This autoregulation is mediated by differential expression of Faim2 and is important for survival. ERK stress-kinase signaling acts upstream of Faim2. Therapeutic interventions targeting ERK pathway to modulate Faim2 expression may be used to prevent photoreceptor apoptosis after retina-RPE separation.
